# Accurate prediction of response to endocrine therapy in breast cancer patients: current and future biomarkers

**DOI:** 10.1186/s13058-016-0779-0

**Published:** 2016-12-01

**Authors:** Cigdem Selli, J. Michael Dixon, Andrew H. Sims

**Affiliations:** 1 Applied Bioinformatics of Cancer, Edinburgh Cancer Research Centre, Institute of Genetics and Molecular Medicine, Edinburgh, EH4 2XR UK; 2 Edinburgh Breast Unit, Western General Hospital, Edinburgh, EH4 2XR UK; 3 Department of Pharmacology, Faculty of Pharmacy, Ege University, Izmir, 35050 Turkey

**Keywords:** Endocrine therapy, Liquid biopsy, Response prediction, Sequential sampling

## Abstract

Approximately 70% of patients have breast cancers that are oestrogen receptor alpha positive (ER+) and are therefore candidates for endocrine treatment. Many of these patients relapse in the years during or following completion of adjuvant endocrine therapy. Thus, many ER+ cancers have primary resistance or develop resistance to endocrine therapy during treatment. Recent improvements in our understanding of how tumours evolve during treatment with endocrine agents have identified both changes in gene expression and mutational profiles, in the primary cancer as well as in circulating tumour cells. Analysing these changes has the potential to improve the prediction of which specific patients will respond to endocrine treatment. Serially profiled biopsies during treatment in the neoadjuvant setting offer promise for accurate and early prediction of response to both current and novel drugs and allow investigation of mechanisms of resistance. In addition, recent advances in monitoring tumour evolution through non-invasive (liquid) sampling of circulating tumour cells and cell-free tumour DNA may provide a method to detect resistant clones and allow implementation of personalized treatments for metastatic breast cancer patients. This review summarises current and future biomarkers and signatures for predicting response to endocrine treatment, and discusses the potential for using approved drugs and novel agents to improve outcomes. Increased prediction accuracy is likely to require sequential sampling, utilising preoperative or neoadjuvant treatment and/or liquid biopsies and an improved understanding of both the dynamics and heterogeneity of breast cancer.

## Background

Breast cancer is the most common cancer in women and second most common cancer worldwide [1]. A heterogeneous and dynamic disease, breast cancer exhibits unique acquired somatic mutations and gene expression changes that underpin the two main mortality factors: disease recurrence and drug resistance. Resistance to endocrine therapy can occur at disease inception (de novo or intrinsic resistance) or develop during treatment (acquired resistance), and various molecular resistance mechanisms have been proposed [2]. Breast cancer is divided into several subtypes with distinct histological, genomic and transcriptomic profiles, outcomes, and responses to therapy [3]. Fundamentally, expression of oestrogen receptor alpha (ER) classifies patients simply as either having ER-positive (ER+) or ER-negative disease, the former representing approximately 70% of patients. However, its expression is heterogeneous and its predictive value is limited since only up to 50–70% of ER+ patients respond clinically to neoadjuvant endocrine therapy [4, 5] indicating the need for biomarkers that predict those likely to respond. In addition, responders exhibit considerable heterogeneity in their responses to endocrine agents and as many as 40–50% of ER+ patients relapse in the adjuvant setting [6]. Whilst adjuvant endocrine treatment reduces 5-year recurrence rates significantly, it has less effect on 5-year mortality rates. For instance, 5 years of tamoxifen treatment reduces mortality by 25% of its relative reduction of recurrences [7].

**Fig. 1:**
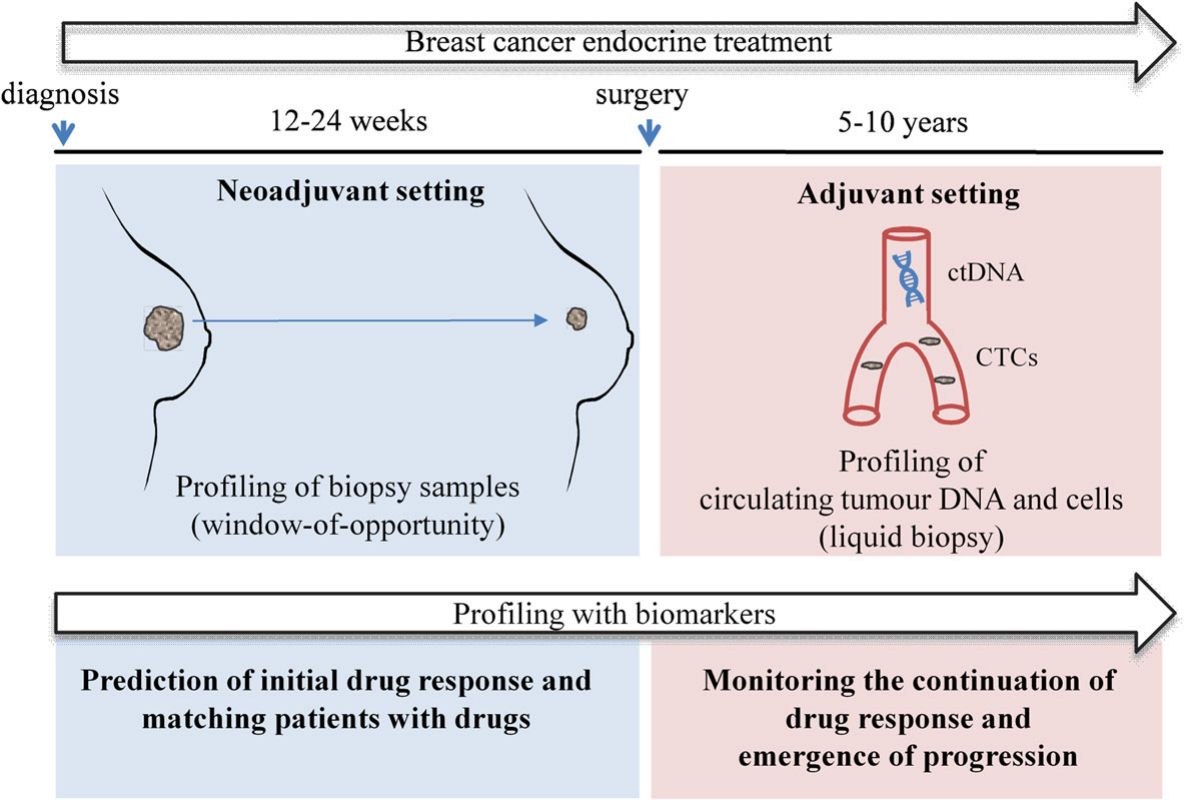
A combined approach for individualised prediction of endocrine treatment response. Profiling of biopsy samples during neoadjuvant drug treatment may help to match drugs to patients and to predict treatment response. Neoadjuvant assessment should be combined with long-term follow-up of individual tumour changes acquired during treatment via non-invasive (liquid biopsy) sampling. Profiling circulating tumour cells (*CTCs*) and circulating tumour DNA (*ctDNA*) can be used to monitor continuous benefit from adjuvant treatment and to detect disease progression. In future practice, a combination of these approaches with predictive biomarkers would provide a more accurate prediction of endocrine therapy response

Recently the use of endocrine therapy in the neoadjuvant setting has allowed clinicians to detect patients that benefit from treatment over a short-term period by simultaneously monitoring tumour volume. In addition to the short-term benefit, neoadjuvant treatment response is reflective of adjuvant benefit [8] and further allows established or novel drugs to be tested, either alone or in combination [9]. This can allow rapid introduction of efficient novel therapies as well as predictive biomarkers into the clinic. Although assessment or biopsy of the primary tumour during treatment may improve response prediction, this approach could be combined with non-invasive long-term follow-up to ensure that treatment benefit continues (Fig. 1). Potential plasma biomarkers such as circulating tumour cells (CTCs) and cell-free DNA may provide similar data on long-term efficacy of endocrine treatments.

In this review, we discuss the current and potential future of endocrine treatment response prediction and biomarkers for individualization of breast cancer therapy in patients with ER+ cancers. Combining endocrine treatment with emerging drugs is also discussed. Current conventional biomarker and drug discovery approaches using heterogeneous patient populations should be replaced with more time-efficient and individualised designs. Monitoring treatment response by comparing before treatment and on-treatment blood and biopsy samples should allow implementation of individualised endocrine therapy.

## Targeted endocrine treatment of breast cancer

Tamoxifen was the first selective oestrogen receptor modulator (SERM) used clinically and is still widely prescribed in pre-menopausal women; it antagonistically regulates ER in breast and brain tissue but is an agonist in the uterus and liver. Aromatase inhibitors (AI), including letrozole, anastrozole, and exemestane, block oestrogen production by inhibition of aromatase in both peripheral tissues and in the cancer, and are effective only for post-menopausal women in whom the major source of oestrogen is peripheral tissues such as adipose tissue and the breast. The selective oestrogen receptor downregulator (SERD) fulvestrant has been shown to be as effective as anastrozole but with a significantly longer time to progression when used as first-line endocrine therapy in post-menopausal women with advanced ER+ breast cancer in the initial analysis of the FIRST trial [10]. Recent randomized studies have demonstrated extended overall survival with first-line [11] and extended progression-free survival with second-line fulvestrant when compared to anastrozole (phase III FALCON trial).

Targeted endocrine treatments for ER+ breast cancer have been available for over 30 years and have had a significant impact in improving mortality rates [7], although inhibiting ER signalling is not effective in all patients with ER+ cancers. Many ER+ breast cancer patients that initially benefit from first-line endocrine therapy go on to relapse as a consequence of the cancer developing endocrine resistance.

## Novel approaches to overcome endocrine treatment resistance

A number of promising agents aimed at overcoming endocrine resistance by targeting signalling pathways are currently under clinical investigation [12]. Detailed information regarding the molecular mechanisms of endocrine resistance can be found in a recent review by Clarke et al. [2].

In ER+ disease, loss of ER expression is not a common resistance mechanism and occurs in only a limited number of patients, and most tumours still respond to second- or third-line anti-oestrogen therapies. More commonly, resistance is facilitated by sequential upregulation of pathways downstream of ERα such as phosphoinositide-3-kinase/AKT/mammalian target of rapamycin (PI3K/AKT/mTOR) signalling. One future aim is to restore sensitivity to endocrine therapy or prevent resistance outright by using combined endocrine agents or combining endocrine agents together with novel drugs. Combinations of current drugs, such as fulvestrant and anastrozole, are being tested in the metastatic setting but the results so far have not clearly shown an advantage for the combination [13, 14], and potentially highlight the need for new drug combinations to be investigated. Other potential candidate combination includes endocrine agents together with agents that target the cell cycle, although the efficacy of this combination depends on the individual molecular profile of the cancer. Enhanced toxicity and the cost of combined therapy are limitations that must be overcome. Given the inherent heterogeneity of ER+ disease, individualising therapy is proving difficult without predictive biomarkers. Whether all patients require the combination is also unclear, although there is optimism that outcomes for patients whose cancers continue to proliferate on endocrine therapy will be improved by these combinations [15].

ER signalling exhibits crosstalk with various growth factor receptor signalling pathways that promote ligand-independent activation of ER. However, current clinical studies do not support the additional clinical benefit of combining endocrine therapy with growth factor inhibitors such as gefitinib [16], bevacizumab [17], and ganitumab in post-menopausal ER+ locally advanced or metastatic breast cancer patients. Although these trials were performed in a specific subpopulation of patients based on current biomarkers and clinical criteria, these subgroups are not homogeneous. It could be that the negative results resulted from patient heterogeneity and that some individual patients did benefit, again emphasising the need for improved biomarkers to predict those who may benefit from specific growth factor inhibitors.

The PI3K/AKT/mTOR pathway is downstream of ER and other growth factor receptor signalling pathways and its hyperactivation has been reported in resistance to both endocrine and trastuzumab therapy [4]. Several agents targeting PI3K and AKT are currently being studied in clinical trials. Mutations in PIK3CA, AKT, and PTEN, an endogenous inhibitor of PI3K signalling that limits cell proliferation, have been suggested to predict response to therapy. In a study we performed, PIK3CA mutation status did not change during development of endocrine resistance and so is not a mechanism of resistance, but mutation status at diagnosis could be used to determine who might benefit from a PI3K inhibitor either as adjuvant or at relapse and development of subsequent metastatic disease [18].

Downstream of PI3K, mTOR appears to be a more promising drug target. The cumulative benefit of combination therapy with letrozole and the first mTOR inhibitor everolimus has been demonstrated using the neoadjuvant setting [19]. Dramatic downregulation of phospho-S6, a downstream effector of mTOR, was observed only in everolimus-treated patients, supporting a potential predictive role of phospho-S6 in response to mTOR inhibitors [19].

Downstream of all the pathways outlined above is the cell cycle machinery. Cyclin D1, which activates cell cycle re-entry via cyclin-dependent kinase (CDK)4 and CDK6 is the transcriptional target of ER and growth factor pathways. Oral selective CDK4/6 inhibitors (palbociclib, ribociclib, and abemaciclib) in combination with endocrine therapies are under investigation [20], and potential predictive markers of the treatment benefit other than ER positivity are also being investigated. In recent clinical trials that demonstrated improved clinical benefit with CDK4/6 inhibitors, cyclin D1 amplification and p16 loss were not significant predictors of benefit, and ER status was the most effective predictive marker in identifying patients likely to respond to first-line CDK4/6 inhibitor therapy [21, 22].

The histone deacetylase (HDAC) inhibitors panobinostat, vorinostat, and entinostat are other new drugs being investigated in ER+ breast cancer. Patients could potentially be stratified by assessing breast cancer-specific HDAC enzyme expression and acetylation levels of target proteins before and after treatment [23].

## Old drugs with new applications

In addition to novel drugs, currently approved drugs with potential anti-tumour activities are being investigated in patients with ER+ disease prevention and treatment. Metformin, used to treat type II diabetes, and statins, used for the treatment of hyperlipidaemia, are examples. Anti-tumour activities of metformin as monotherapy [24] and its synergistic potential in combination with neoadjuvant letrozole [25] are currently being studied. Although the anti-tumour mechanism of action of metformin is not yet clear [24], its anti-tumour effects in breast cancer are suggested to be via an indirect pathway by decreasing both insulin plasma levels and inhibiting downstream PI3K and mitogen-activated protein kinase (MAPK) signalling. Based on the results from a recent window-of-opportunity study, serum insulin, tumour insulin receptor expression, p-Akt, and Ki-67 are potential biomarkers of tumour metformin sensitivity [26].

Stable upregulation of cholesterol biosynthesis pathways was recently determined in AI-resistant cells [27]. This is consistent with epidemiological data showing that patients with ER+ cancers who have high expression of cholesterol biosynthesis pathways at diagnosis have shorter recurrence- and metastatic-free survival, thus suggesting a predictive role for cholesterol biosynthesis pathways in endocrine treatment response [27]. One study with a population of patients with a mix of ER+ and ER-negative cancers failed to show any correlation between response to simvastatin and selected drug- and breast cancer risk-specific biomarkers, serum concentrations of high-sensitivity C-reactive protein, oestrogens, and fasting lipids [28]. Despite this, further trials of specific subgroups of patients may identify groups who benefit from this treatment and perhaps also predictive biomarkers.

Based on the promising preclinical data [29], anti-resorptive bone agents such as bisphosphonates and donesumab are under clinical investigation in metastatic breast cancer. A recent meta-analysis of clinical trials shows that adjuvant bisphosphonates improve breast cancer survival by reducing breast cancer recurrence in the bone in post-menopausal women but not pre-menopausal women [30], possibly due to differential bone turnover rate and differences in bone microenvironment before and after the menopause. Bone turnover markers released during bone resorption, such as the carboxyterminal peptide of type I collagen, may predict which patients are at increased risk of bone relapse [31] as well as allowing monitoring of response to anti-resorptive therapy.

**Fig. 2:**
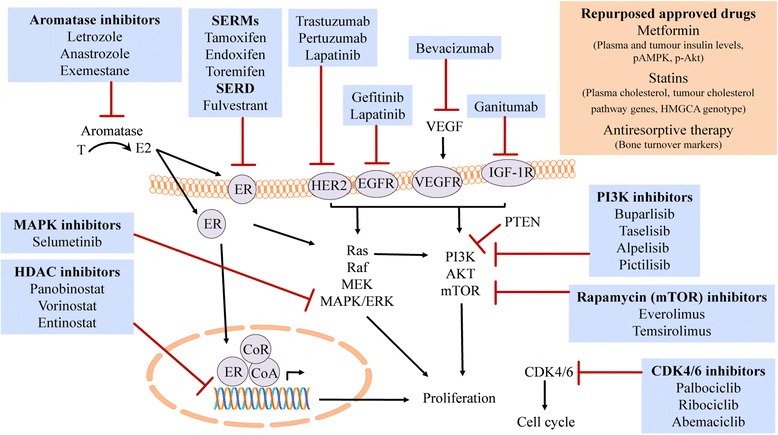
Drugs used in endocrine treatment of breast cancer. Schematic diagram of current and novel drugs currently in clinical trial with their target signalling molecules. *CDK* cyclin-dependent kinase, *E2* oestradiol-17 beta, *EGFR* epidermal growth factor receptor, *ER* oestrogen receptor, *HDAC* histone deacetylase, *HER2* human epidermal growth factor receptor, *IGF-1R* insulin-like growth factor-1 receptor, *MAPK* mitogen-activated protein kinase, *mTOR* mammalian target of rapamycin, *PI3K* phosphoinositide-3-kinase, *SERD* selective oestrogen receptor degrader, *SERM* selective oestrogen receptor modulator, *T* testosterone, *VEGF* vascular endothelial growth factor, *VEGFR* VEGF receptor

In the future, novel drugs targeting critical molecular pathways that exhibit crosstalk with ER will likely be used in combination with endocrine therapy based on tumour-specific profiles. Real-time monitoring of treatment response may allow cancers that develop acquired mutations in currently druggable targets to be identified and allow patients to alter their treatment as appropriate. Combinations of established and novel drugs currently in clinical trials with their associated target molecules are summarised in Fig. 2. Predicting which patients will benefit from these combinations will be a major challenge.

## Current predictive biomarkers of endocrine treatment

Three biomarkers, ER, progesterone receptor (PR), and human epidermal growth factor receptor 2 (HER2/ErbB2), are currently measured both for diagnosis of disease subtype and as a guide to treatment. ERα presence is the best predictor for endocrine treatment; however, patient response remains heterogeneous. It is known that the ESR1 levels correlate with treatment outcome, with low levels of ESR1 mRNA in the primary tumour being associated with decreased tamoxifen benefit [32]. PR expression, which is downstream of ER signalling and regulated by ER, is associated with good prognosis, although its predictive role in endocrine therapy response remains unclear. Benefit from tamoxifen is similar in both PR-positive and PR-negative patients [7]. On the other hand, the presence of PR and quantitative levels have been shown to correlate significantly with time to AI treatment failure in ER+ patients, suggesting its predictive role in AI response [33]. However, PR status could not differentiate the relative benefit of anastrozole over tamoxifen [34]. A functional crosstalk between PR and ER, decreased PR expression linked with altered ER chromatin binding and poor clinical outcome, has been reported recently [35]. The third routinely used biomarker, HER2, is a predictor of adverse outcome in patients receiving adjuvant endocrine treatment. Not all patients with ER+ HER2+ cancers relapse, and responses to HER2-directed therapy are much less frequent in ER+ HER2+ cancers than ER– HER2+ cancers [36]. Nonetheless, a subgroup of patients with ER+ cancers that overexpress HER2 are candidates for targeted anti-HER2 therapy such as trastuzumab, pertuzumab, and lapatinib in combination with chemotherapy.

## Multigene signatures predict prognosis

Besides these well-established immunohistochemical markers, an RNA-based multigene test that directly predicts patients who will effectively respond to endocrine therapy has not yet made it to routine clinical practice. Using individual patient-derived genomic information to predict benefit is the underlying goal of individualised therapy. This should allow matching the right drug with the right patient. Microarray-based gene expression analysis of cell lines and patient-derived primary samples has been widely used to find drug response-related gene signatures. Comparing different profiling approaches, microarrays have been shown to be the most powerful in predicting drug sensitivity in human breast cancer cell lines (NCI-DREAM project) [37]. Advances in genomic technology and bioinformatics analysis have led to the molecular sub-classification of breast cancer with prognostic implications and introduction of multigene assays into the clinic. Based on gene expression analysis, ER+ breast cancer has been sub-classified into two main categories—luminal A and luminal B—the latter associated with a poorer outcome [38]. Multigene expression assays that are currently available in the clinic also help to predict prognosis. For instance, the Food and Drug Administration (FDA)-approved Prosigna breast cancer gene signature assay, based on the PAM50 intrinsic subtype classification model [39], provides assessment of 10-year risk of distant recurrence of post-menopausal ER+ patients. In addition, other multi-gene expression tests such as Oncotype DX (21-gene signature), MammaPrint (70-gene signature), and EndoPredictClin (a quantitative RT-PCR-based assay of eight genes) are helpful in identifying a subpopulation of ER+ patients with a low recurrence risk who can potentially avoid chemotherapy or benefit from extended adjuvant hormonal therapy. Recently, the clinical utility of Oncotype DX has been validated prospectively by identifying low-risk patients with ER+ HER2– cancers who do not benefit from adjuvant chemotherapy [40]. Although the multigene assays available today are useful in predicting prognosis in terms of relapse and risk stratification, their ability to predict endocrine treatment efficacy has not been validated. To increase response prediction accuracy using array profiling, dataset and sample composition (i.e. non-tumour content) should also be considered [41, 42].

## Is accurate prediction possible?

Whilst prognostic factors correlate with expected disease course, a predictive biomarker is associated with response to a particular therapy and provides information on the likely benefit from that treatment. Some biomarkers can be both predictive and prognostic, such as ER status and HER2 amplification. In addition, a biological tumour characteristic can both be a therapeutic target and biomarker.

For clinical evaluation of cancer therapeutics, assessment of tumour burden and disease progression are currently used as endpoints in clinical trials. To standardize the evaluation criteria used in clinical studies, the Response Evaluation Criteria In Solid Tumours (RECIST) guidelines were published [43]. Assessment of tumour volume reduction using imaging can be an objective but sometimes insensitive method because image resolution may not be good enough to allow accurate measurements to be obtained. Tumour burden is commonly assessed uni-dimensionally by mammography and ultrasonography, although volumetric or functional imaging tools such as magnetic resonance imaging (MRI) may be more accurate. MRI is recommended over mammography and computerised tomography in assessing response in the neoadjuvant setting [43]. The definition and assessment of outcomes in trials vary and includes time-to-progression, progression (recurrence/disease/relapse)-free survival, and overall survival, making direct comparison of results from different trials difficult.

One challenge in identifying predictive biomarkers using conventional clinical trial design is the necessity for long-term follow-up data. The neoadjuvant setting offers a potential solution to this problem by allowing relatively quick detection of patients that benefit from an intervention [44]. The accepted method of assessing response to neoadjuvant chemotherapy response is pathological complete response (pCR) rate defined by the absence of residual tumour burden in the breast and associated axillary lymph nodes. Pertuzumab received an accelerated FDA approval based on meeting this criterion, in the absence of long-term data in the neoadjuvant setting [9]. In a pooled analysis of neoadjuvant clinical trials, pCR was shown to correlate with long-term clinical benefit [45]. However, correlation of pCR with improved survival benefit was most evident in triple-negative disease and HER2-positive cancer, but was not significant for patients with ER+ low-grade cancers [45]. Also, in practical terms, the molecular evaluation of response is not feasible in pCR patients due to the unavailability of tumour material following chemotherapy.

A further limitation of current clinical studies is the use of pooled data analysis obtained from large heterogeneous groups of patients. Even if clinical studies are performed using specific patient subgroups, such as only ER+ and HER2-negative patients, high degrees of molecular heterogeneity are still evident. The Signature Clinical Trial Program, a modular phase II trial by Novartis, aims to overcome drawbacks of traditional clinical trials by linking targeted therapies to patients with pathway-activated tumours. Further trials using specific patient subgroups with specific mutations to target genes such as ER and PI3K may help to more accurately identify predictive biomarkers. In addition to inter-patient heterogeneity, intra-tumour heterogeneity is another obstacle in effective breast cancer treatment and response prediction. Breast cancers can be composed of a dominant clone that represents the primary tumour with minor quiescent subclone(s) [46]. The only way to predict individual response to therapy is the real-time profiling of tumour samples from individual patients because of such a heterogeneous and evolving disease profile. There is a real need to determine novel biomarkers on an individual basis that differentiate patients that are most likely to respond or acquire resistance to endocrine therapy. Accurate prediction is not possible in the absence of biomarkers derived from the individual characteristic of every tumour.

## The neoadjuvant setting to predict endocrine treatment response

Short-term pre-operative trials with specific groups of patients have proved promising in identifying predictive biomarkers of the efficacy of anti-cancer-targeted therapies [47]. Early evidence of endocrine drug effectiveness can be obtained in the pre-operative (neoadjuvant) setting by profiling baseline and on-treatment biopsy samples utilising the window-of-opportunity. This predictive evidence acquired during short-term neoadjuvant therapy can help to identify individual patients that will potentially benefit from long-term adjuvant treatment enabling personalized approaches. In the following section, we will summarise the current neoadjuvant studies that have investigated prediction of endocrine treatment response.

The largest window-of-opportunity trial to date includes over 4000 post-menopausal ER+ patients; POETIC (Peri-Operative Endocrine Therapy for Individualizing Care) is currently in its follow-up period [48]. In POETIC, patients were randomised to receive either no treatment or 2 weeks of treatment of an AI both before and after surgery with one aim being to determine biomarkers of response and resistance to oestrogen deprivation. The clinical predictive value of on-treatment proliferation biomarker Ki-67 levels for long-term outcome is also being assessed in this trial. Although short-term reduction in Ki-67 can predict clinical response to endocrine treatment [49], the accuracy of prediction should be increased using additional biomarkers. However, before the introduction of Ki-67 measurement in clinical practice as an early surrogate marker of treatment efficiency, further studies for the standardization of measurements, ensuring reproducibility and consistent scoring, are needed [50, 51]. It will be several years before the trial matures sufficiently to fully evaluate how current or potential on-treatment markers/signatures relate to long-term outcomes.

In a recent window-of-opportunity study, proliferation genes after treatment were able to accurately predict AI response at 2 weeks, but not before treatment [52]. Furthermore, a four-gene signature measuring two genes pre-treatment and two genes after 2 weeks has recently been shown to predict accurately clinical response to 3 months of neoadjuvant letrozole treatment in ER+ patients. The test was also predictive for relapse-free and breast cancer-specific survival [52]. The genes that predicted response to AI included two pre-treatment genes associated with immune response (IL6ST) and apoptosis (NGFRAP1) and two genes measured after 2 weeks of treatment associated with proliferation (MCM4 and ASPM). Changes of gene expression in response to neoadjuvant letrozole treatment were similar in both invasive ductal and lobular carcinoma [53]. These observations suggest that transcriptomic changes that develop during treatment are representative of the drug’s mechanism of action and independent of the type of tumour treated, suggesting that suppression of proliferation is the main driver of response. If molecularly and histologically distinct subtypes are responding in the same way, then this finding adds to the idea that pre-treatment differences alone may not define response to treatment, and that on-treatment changes may be more important.

In a recent neoadjuvant phase II trial with a sequential biopsy design, the CDK4/6 inhibitor palbociclib given in combination with anastrozole was shown to be more anti-proliferative than anastrozole alone by using sequential Ki67 analysis in ER+ cancer [54]. Effects of another CDK4/6 inhibitor, abemaciclib, in combination with anastrozole is also under investigation in a clinical window-of-opportunity study with evaluation of change in Ki67 expression from baseline to 2 weeks (NeoMONARCH phase II trial). Ribociclib in combination with letrozole for 24 weeks as neoadjuvant endocrine therapy is also being investigated (FELINE phase II trial).

The transcriptional response to fulvestrant has also been investigated in a neoadjuvant study, where a subset of genes was identified with potential predictive utility to differentiate fulvestrant response from oestrogen deprivation by anastrozole [55]. Transcriptional differences overlapped with the drugs’ distinct mechanisms of action. In addition to inhibition of common oestrogenic ER activity, fulvestrant was shown to antagonise oestrogen-independent ER activity with significantly differentiated genes in ER, androgen receptor (AR), and TP53 signalling.

## Long-term monitoring of endocrine treatment response

Although analysing single or sequential breast tumour biopsy samples may help to estimate individual responsiveness before starting treatment, it is not likely that they will predict continued response or emergence of resistance. Another concern of sampling the tumour only at diagnosis is that the sampled portion may not reflect any intra-tumour heterogeneity [46], a major challenge to successful and individualised cancer therapy. Repetitive and non-invasive sampling and analyses of CTCs can help to implement personalized treatments by monitoring drug response in metastasis-prone breast cancer patients.

It is suggested that drug-resistant subclone(s) may be present at diagnosis or may emerge during the treatment leading to breast cancer metastasis [56]. Functional relevance of the intra-tumour heterogeneity in ER+ breast cancer has been demonstrated in a number of recent studies. In ER+ metastatic patients, two somatic mutations within the ligand-binding domain of ER were determined and these mutations were not detected in primary or treatment-sensitive tumours indicating their emergence during endocrine treatment [57]. Acquired ESR1 mutations detected in metastatic advanced breast cancer patients that are primarily found in ligand-binding domain and leading to constitutive ER activity have been recently reviewed [58]. Although their role in metastasis has not yet been confirmed, resistance-associated mutations may be predictive markers and also potential new targets in metastatic disease.

Whether they are present at the diagnosis or emerge under the pressure of treatment, resistant clones need to enter the circulation to cause disease progression. Based on this, monitoring of CTCs that will eventually give rise to recurrences and circulating tumour DNA (ctDNA) may help to overcome therapeutic failure through emergence of resistant clones.

## Circulating tumour cells

Recently, the clinical utility of CTC count in prognostication of metastatic breast cancer patients has been analysed by pooling of data from 1944 patients and 20 studies [59]. Both baseline CTC count and decline at 3–5 weeks after the start of treatment improved survival prediction when added to the clinicopathological models, confirming the independent prognostic effect of CTC count on progression-free survival and overall survival. In addition to its prognostic value, CTC count was also proposed to be an indicator of chemotherapy and endocrine therapy efficacy in metastatic breast cancer patients [60]. Determination of treatment response regardless of drug used is possible with monitoring real-time changes in CTC count.

In addition to CTC count, molecular profiling of CTCs may provide predictive signatures in patients with breast cancer. Characterization of CTCs using different molecular assays such as whole-genome analysis may shed light on tumour heterogeneity and therapy resistance [61]. However, there are some limitations to be overcome before their introduction as clinical tools. In addition to a lack of standardized methods for their harvest, another limitation is that some of the surface markers used to isolate CTCs that are already in low concentrations in plasma, such as E-cadherin, have been shown to be downregulated during epithelial-mesenchymal transition (EMT) of breast cancer [62], making their detection more difficult. Furthermore, mesenchymal cells were shown to be highly enriched in CTCs in contrast to primary tumours [63], indicating the importance of taking account of heterogeneity of CTCs in the therapeutic follow-up of metastatic breast cancer. Current CTC capture methods based on epithelial markers may have limited clinical value since they cannot detect malignant mesenchymal CTCs. The only FDA-approved CTC detection system, the CellSearch® CTC Test, counts only epithelial (EpCAM-positive) CTCs indicating the need for novel technologies with improved clinical utility allowing detection and characterisation of different CTCs subpopulations such as CTCs in EMT [64]. The heterogeneity of mutational status of CTCs isolated from the same breast cancer patient [65] also indicates the necessity of characterizing CTCs at the single-cell level.

## Circulating tumour DNA

Screening cell-free ctDNA released from dying tumour cells into the bloodstream is another potential, non-invasive way to monitor the progression of cancer. ctDNA and CTCs are different in their sensitivity; ctDNA has been suggested to be a more sensitive measure for response prediction in metastatic patients [66]. In addition, ctDNA was present in the plasma of patients without any detectable CTCs and they were also detectable in half of patients with localised breast adenocarcinoma [67]. On the other hand, ctDNA and CTCs give different information regarding the primary and metastatic tumour cells. CTCs provide an overall picture of cancer cells at the RNA, DNA, and protein levels, whereas the detection of genetic and epigenetic alterations including acquired mutations in cancer DNA is possible by analysing ctDNA.

One outcome of the ongoing PIKTAM study, investigating buparlisib in combination with tamoxifen, is to validate a technology for sensitive and specific detection of mutations in circulating free tumour DNA that may help towards the establishment of a clinical role for ctDNA. Mutation tracking using serial follow-up ctDNA samples may identify early breast cancer patients with high relapse risk in addition to the use of this genetic information of residual tumour for tailoring of adjuvant therapy [68]. Monitoring ESR1 mutations using ctDNA potentially allow the discontinuation and replacement with other treatments of ineffective endocrine therapies and the prevention of metastatic disease [69]. For instance, serial analysis of ctDNA of a patient revealed that ESR1 mutation was detected following subsequent AI treatment, suggesting selection of mutant clones during metastatic therapy, but PIK3CA mutation was already present before drug exposure [70] which may help to select metastatic patients for ESR1 mutation-targeted therapy. The BOLERO-2 trial demonstrated that ESR1 mutations, which promote ligand-independent receptor activation and resistance to endocrine therapy, were prevalent in ctDNA from baseline plasma samples of AI-treated ER+ metastatic patients and were associated with shorter overall survival [71]. Advanced breast cancer patients with baseline ESR1 mutations treated with fulvestrant have been shown to have improved outcomes compared with exemestane [72]. However, in a recent retrospective study, serial analysis of ctDNA did not show any evidence for accumulation of ESR1 mutations during fulvestrant treatment, and progression-free survival was not different in patients with ESR1 mutations [73]. Further studies are needed to determine the heterogeneity and potential clinical significance of ESR1 mutations following different drug therapies for better stratification of patients with advanced disease.

In addition to mutational changes, the methylation status of ctDNA may help to monitor treatment efficacy in breast cancer. Although data for neoadjuvant endocrine therapy are not available yet, a number of studies have reported the role of ctDNA in monitoring response to neoadjuvant chemotherapy. Total methylation of a panel of five genes, BRCA1, MGMT, GSTP1, Stratifin, and MDR1, was significantly correlated with tumour volume reduction in responders to neoadjuvant chemotherapy [74]. Also, in patients with methylation in the promoter region of RASSF1A before therapy, methylated ctDNA has been shown to be decreased early following neoadjuvant chemotherapy in responders, but non-responders had persistent methylation levels [75, 76].

The data obtained from circulating blood biomarkers and before the initiation of metastasis will be more valuable than the data from metastatic tumours in the management of metastasis, because the detection and analyses of CTCs and ctDNA with metastasis-initiating potential in different sites will provide more comprehensive real-time data. Therefore, CTCs and ctDNA have the potential to enable prevention of disease recurrence in addition to individualised treatment of metastatic patients. Although their clinical relevance in breast cancer has not been established, there are large number of ongoing clinical trials and growing evidence supporting their role in personalized treatment decisions. Drugs currently in the clinic are mainly anti-proliferative and suppress proliferation of subclones. Identification of new pathways using blood biomarkers may lead to new, more efficient treatments for metastatic disease.

## Future perspectives

Recent studies offer hope that sequential analysis of breast cancers from patients will lead to more accurate predictions of which patients will benefit from treatment with existing, combinations, or enhanced endocrine agents. As tumour cells evolve over the course of treatment, we should also evolve our treatment approaches based on mutational and expressional changes acquired by individual tumours over time. Expansion of studies of neoadjuvant treatment and monitoring CTCs offer great promise in predicting response to treatment and disease progression, and should allow optimal clinical management of emergence of treatment resistance. After overcoming the associated technical difficulties, their clinical utility in monitoring continued endocrine response can be realised.

## Conclusions

Future endocrine treatment response prediction tools will necessitate integration of a wide range of molecular information, including gene expression and acquired mutations, each of which are able to affect drug response. We suggest that a combined approach including biopsy profiling in the neoadjuvant setting as well as liquid biopsy profiling should be used to match effective drugs with individual patients and to monitor continuation of drug effectiveness.

## Abbreviations

AI: Aromatase inhibitors
CDK: Cyclin-dependent kinase
CTC: Circulating tumour cell
ctDNA: Circulating tumour DNA
EMT: Epithelial-mesenchymal transition
ER: Oestrogen receptor alpha
FDA: Food and Drug Administration
HDAC: Histone deacetylase
HER2/ErbB2: Human epidermal growth factor receptor 2
MAPK: Mitogen-activated protein kinase
MRI: Magnetic resonance imaging
pCR: Pathological complete response
PI3K/AKT/mTOR: Phosphoinositide-3-kinase/AKT/mammalian target of rapamycin
PR: Progesterone receptor
SERD: Selective oestrogen receptor downregulator
SERM: Selective oestrogen receptor modulator
